# Emergency department use during pregnancy by Medicaid type

**DOI:** 10.21203/rs.3.rs-5433292/v1

**Published:** 2024-12-12

**Authors:** Alyssa Hersh, Ann Martinez Acevedo, Ava Mandelbaum, Esther Choo, Maria Rodriguez

**Affiliations:** Oregon Health & Science University; Oregon Health & Science University; Oregon Health & Science University; Oregon Health & Science University; Oregon Health & Science University

**Keywords:** emergency department, obstetrics, Medicaid, pregnancy outcomes, health policy

## Abstract

**Background:**

Emergency department (ED) use is common among patients with Medicaid insurance during pregnancy. However, it is unknown how ED utilization differs among those with different types of Medicaid such as Emergency Medicaid, with which access to outpatient care is more restricted.

**Objective:**

We sought to compare differences in ED use during between pregnant persons with Emergency Medicaid and Traditional Medicaid and pregnancy outcomes by ED utilization.

**Study Design:**

This was a retrospective cohort study of all births among Medicaid recipients in South Carolina from 2010 to 2019. The main comparator was type of Medicaid. Our primary outcome was an ED visit during pregnancy. Secondary outcomes included average number of visits, perinatal outcomes, and prenatal and hospital charges.

**Results:**

There were 240,597 births that met inclusion criteria for this analysis. Over the study period, the proportion of patients with at least one ED visit increased for all groups. A higher proportion of patients with Traditional Medicaid had at least one ED visit compared with Emergency Medicaid (58.2% versus 22.7%). Patients who had at least one ED visit were more likely to be younger, of Black race, live rurally, nulliparous, have lower or higher body mass index, and have a higher prevalence of pre-existing medical co-morbidities.

**Conclusion:**

We found that individuals with Traditional Medicaid were more likely to have an antenatal ED visit than individuals with Emergency Medicaid.

## Introduction

Nationally, use of the emergency department (ED) for both urgent and non-urgent reasons is increasing, including during pregnancy.^1,2^ Use of the ED for non-urgent reasons has important safety, quality and economic consequences: ED resources are limited, and should be conserved for medically acute patients.^1,3^ Multiple factors are likely driving the increase in non-urgent ED use, including diffculty accessing primary care and lack of an alternative to receive timely care.^4,5^ While existing research has found conflicting results on whether specific patient characteristics are associated with higher rates of ED use, numerous studies have identified higher ED utilization among people with Medicaid insurance.^6–8^

Over time, the incidence of maternal morbidity has increased concurrently with the medical complexity of the pregnant population.^9^ Epidemiologic studies consistently show that Medicaid insurance type is associated with worse pregnancy outcomes.^9,10^ Pregnancy and its associated complications are leading reasons for ED visits among reproductive age people.^11,12^ The increase in ED use over time may be a natural consequence of this increasing medical complexity of pregnant persons, but also may signify ongoing gaps in access to consistent outpatient or preventive care among certain populations.

Medicaid is the largest payor for births in the US; its policies have a marked influence on maternal health. Medicaid is a joint federal-state insurance program, which provides health care coverage for specific benefits for individuals who meet financial criteria. Pregnancy care, from conception through 60 days postpartum is a covered benefit. However, coverage is not even within Medicaid. For individuals who meet the financial criteria for Medicaid but are not citizens of the United States, coverage is provided through Emergency Medicaid, under which only the hospital admission for birth is covered. States can choose to extend coverage for prenatal or postpartum care using federal waivers, or state-only funds, however many states have not exercised these options.^13,14^

Studies examining ED use during pregnancy typically have analyzed Medicaid policies in general and have not examined the role that variations in Medicaid coverage, such as Emergency Medicaid, may play in influencing rates of ED use during pregnancy.^12,15^ We used ten years of Medicaid claims data from South Carolina, a state that covers only a hospital admission for birth for all Medicaid recipients, to examine ED utilization among Emergency Medicaid recipients compared to Traditional Medicaid beneficiaries (those who are US citizens with standard Medicaid coverage). We examined ED visits over time, reasons for ED use, and the costs of ED care by type of Medicaid. We estimated the association between type of Medicaid and an ED visit.

## Materials and Methods

We conducted a retrospective cohort study of births among Medicaid recipients in South Carolina between October 1, 2010 through December 31, 2019. Our data included Medicaid claims linked with birth certificate and hospital discharge data. Inclusion of hospital discharge data allowed us to examine all ED visits, regardless of whether they were reimbursed by insurance or not. All data were obtained from the state of South Carolina under a data use agreement. The datasets were fully deidentified prior to transfer to the study team; informed consent was therefore not obtained. We followed the Strengthening the Reporting of Observational Studies in Epidemiology reporting guideline.^16^ This study was approved by the Institutional Review Board at Oregon Health & Science University.

Our primary exposure variable was Medicaid type at birth, Emergency or Traditional. In South Carolina, Traditional Medicaid covered outpatient and inpatient pregnancy care, from conception through 60 days postpartum for individuals with income up to 194% of the federal poverty level (FPL). Emergency Medicaid covered the hospital admission for childbirth, and any care for a life-threatening condition, for individuals meeting the same financial criteria, but not the US citizenship requirement.

Our primary outcome was an ED encounter during pregnancy. Our independent variable was type of Medicaid, Emergency or Traditional. We identified ED encounters using hospital discharge data, which allowed us to include all ED encounters, including visits that were not reimbursed by an insurer. The ED encounter data included visits covered by Medicaid, Commercial Insurance, and Charity Care, as well as self-pay encounters. Charity Care were visits for Medicaid patients that were not reimbursed by insurance and written off by the hospital. Self-pay encounters were for visits by Medicaid patients that were not reimbursed by Medicaid, and the individual was billed.

From the hospital encounter data, we created two binary outcome variables: any ED visit during pregnancy, and any ED visit during pregnancy that was covered by Medicaid. We captured the total number of ED visits during pregnancy per birth and categorized those with an ED visit in pregnancy as either all self-pay and/or charity care, or at least one or more visits covered by Medicaid or another insurance. We also summarized the average total charges per ED visit per person.

We abstracted demographic and clinical information from the birth certificate files and claims data. We included the demographic variables of maternal age (< 20, 20–24, 25–34, and ≥ 35 years), multiparity, race and ethnicity, county of residence (metropolitan, nonmetropolitan, missing), and state. We included the clinical variables of adequate prenatal care (≥ 7 prenatal visits), mode of delivery, preterm birth (births < 37 weeks’ gestation), and pregnancy complications (preexisting diabetes, preexisting hypertension, gestational diabetes, and gestational hypertensive disorders).

As differences in medical complexity may explain ED use, we assessed whether medical complexity varied by type of Medicaid through calculating rates of severe maternal morbidity (SMM) and the Obstetric Comorbidity Index (OCI) score using International Classification of Disease (ICD) codes. From Medicaid claims data, we identified intrapartum SMM, including blood transfusion using the Center for Disease Control definition.^17^ We categorized a birth event as experiencing SMM if at least one International Classification Diagnosis (ICD) (versions 9 and 10) code was present on the childbirth hospitalization claims. To more broadly capture high risk pregnancies, we also calculated the OCI score using the maternal childbirth hospitalization claims.^18^ We only used childbirth hospitalization claims because prenatal care is not covered for EM recipients in South Carolina. We summarized the risk score on the person-birth level. We reported the OCI as both a continuous measure and a binary variable. We categorized a “high” OCI by identifying the cutoff of risk score that produced a 50% sensitivity of SMM (i.e., among those with intrapartum SMM, 50% had a risk score at or above a specific threshold). In our overall study population, a risk score at or above 11 was considered “high” OCI.

## Statistical analysis

We used descriptive statistics to determine the proportion of births with any ED visits during pregnancy by Medicaid type and payor (Medicaid vs Self-pay/Charity Care). We assessed for trends over time using linear regression.

We then restricted our sample to births with at least one ED visit during pregnancy and examined obstetric outcomes, mean number of ED visits during pregnancy, and median cost per ED visit by Medicaid Type and Self-pay/Charity Care. We evaluated the top five primary diagnoses associated with an ED visit by type of Medicaid. We estimated the association between Medicaid Type and binary ED visit outcomes using multivariable logistic regression. We selected confounders using a causal modeling approach. Our models adjusted for maternal age and attending at least five prenatal care visits. All analyses were conducted using a p-value of 0.05 and using R software.

## Results

**Figure 1:**
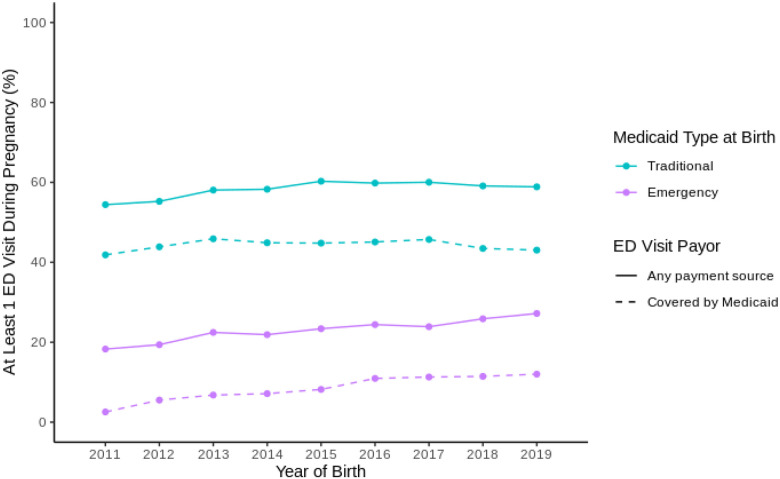
Proportion of South Carolina Medicaid Births with an Emergency Department (ED) Visit During Pregnancy by Medicaid Type at Birth and ED Visit Payor

Our final analytic sample included 240,597 births. Overall, 55.1% of Medicaid recipients had at least one visit to the ED during pregnancy (Supplemental Table 1). Over half of patients (58.2%) with Traditional Medicaid had at least one ED visit compared with less than a quarter (22.7%) of those with Emergency Medicaid (Supplemental Table 1). This pattern remained qualitatively similar across our study period (Fig. 1). Pregnant persons who had at least one ED visit were more likely to be younger, of Black race, live rurally, nulliparous, and have lower or higher body mass index; there were higher proportions of numerous medical co-morbidities among those that had at least one ED visit, including tobacco use, chronic and gestational hypertension, and pre-pregnancy diabetes (Supplemental Table 1).

Among Medicaid enrollees with at least one ED visit (n = 132,630), Emergency Medicaid enrollees were on average older (16.4% vs 5.4% ≥35 years old), Latina (57.0% vs 1.3% Hispanic/Latina), and more likely to live in urban areas (70.4% vs 59.9% urban) than their Traditional Medicaid counterparts (Table 1). Emergency Medicaid recipients also differed on important clinical characteristics; they were less likely to receive prenatal care (prenatal visit mean 0.8, SD 1.2 vs 5.3, SD 3.2), less likely to smoke or have hypertension, and more likely to have diabetes (Table 1). While we did not observe a difference in rates of intrapartum SMM between the two groups, the mean obstetric comorbidity index score for Emergency Medicaid recipients was lower than Traditional Medicaid recipients (7.4% vs 8.2%).

The top primary diagnoses for ED visits appeared qualitatively similar by type of Medicaid with “unclassified complications of the antepartum,” bleeding and infection being common causes (Supplementary Table 2). However, the financial implications for individuals of an ED visit varied markedly by type of Medicaid, despite similar diagnoses for seeking care. Among Emergency Medicaid recipients who had an ED visit, 58.4% of visits were not reimbursed by insurance, as compared with 8.4% of Traditional Medicaid recipients. The median cost of an uncovered ED visit was $2,330 among Emergency Medicaid recipients, as compared with $1,780 for a Traditional Medicaid beneficiary (Table 2).

Our regression model confirmed our bivariate findings (Table 3). Emergency Medicaid patients had significantly decreased odds of using the ED during pregnancy as compared with Traditional Medicaid recipients (aOR 0.26 95% CI 0.25–0.27). Emergency Medicaid recipients were also significantly less likely to have their ED visit covered by insurance (aOR 0.14 95% CI 0.13–0.15).

## Comment

### Principal Findings

In this study of Medicaid recipients in South Carolina, we found that while overall ED visits among pregnant individuals with Medicaid insurance were very common, Emergency Medicaid recipients were significantly less likely than those with Traditional Medicaid to visit the ED during pregnancy. This finding may be partially explained by costs of care, particularly related to lack of coverage for non-delivery hospitalization pregnancy care. Emergency Medicaid enrollees were significantly less likely to have their care covered by Medicaid despite qualitatively similar discharge diagnoses.

Even though Emergency Medicaid recipients were older and less likely to receive prenatal care, they visited the ED less than Traditional Medicaid recipients. Furthermore, the mean obstetric comorbidity index score for Emergency Medicaid recipients was lower than Traditional Medicaid recipients, suggesting this lower use of the ED was not associated with worse perinatal outcomes. Given reasons for ED visits were similar between groups, these results suggest an excess of visits for Traditional Medicaid recipients without reduction in adverse outcomes.

### Results in the context of what is known

Childbirth is a common cause of medical debt in the United States.^19,20^ Our study demonstrates that Emergency Medicaid recipients, who are majority Latina, are significantly more likely to have medical bills and debt than Traditional Medicaid peers. Previous research has established the impact that medical debt has on early childhood health and development.^21^ Among families having trouble paying medical bills, children have increased odds of delaying needed health care and having unmet health needs.^21^ Medical debt increases the odds of food insecurity among families, which is associated with poor physical and mental health, and academic performance.^22,23^ Our finding, of increased medical debt among Emergency Medicaid recipients may impact the social determinants of health of their citizen children.

One notable difference between Medicaid types was the difference in hospital charges associated with ED visits. Although the average number of visits was higher among those with Traditional Medicaid, the average charges associated with the ED visit were higher among those with Emergency Medicaid. Given our study design, we were unable to elucidate what contributed to these increased costs, and this should be a topic of future investigation.

### Clinical Implications

If states choose a policy of just covering the delivery hospitalization, it discourages prenatal care, a key access point at which downstream pregnancy complications can potentially be prevented. Studies have shown that expanding coverage of pregnancy care for underinsured people improves provision of care and detection of pregnancy complications.^24–27^ Without access to routine outpatient care or a provider to help triage ED visit necessity, the onus is on the pregnant person to decide whether a symptom or condition merits a costly trip to the ED.

Despite advances in obstetric care, maternal morbidity and mortality is worsening. Regardless of prenatal care access, there will always be need for the ED to identify critical pregnancy-related issues. This means that ED providers must be equipped to care for pregnant patients, either through ensuring appropriate education and training for ED clinicians. A proposed solution has been the implementation of Obstetric EDs, although they may be less accessible and hard to disseminate.^28^ Similarly to what we found in our study, prior studies have shown that pregnant patients who have ED visits are more likely to have worse outcomes, highlighting the opportunity to identify those at high risk for adverse pregnancy outcomes for possible intervention.^29,30^ Any interventions aimed to reduce ED utilization must not do so at the expense of early identification of serious health conditions.

### Research Implications

Our study expands on previous literature by examining ED usage during pregnancy among Emergency Medicaid recipients, an understudied population. We found a much lower rate of ED usage during pregnancy than has been described in the Traditional Medicaid population.^12,15^ Our study demonstrates that patient characteristics, comorbidities and outcomes differ by Medicaid type in the setting of ED visit utilization, suggesting that target programs to reduce ED utilization should account for differences in characteristics of patients who present to the ED. While interventions to reduce ED utilization have been studies in other areas of medicine, this has not been well studied in a pregnant population. Future studies should assess evidence-based education initiatives and programs to assess whether ED visit utilization can be safely decreased without worsening patient outcomes.

### Strengths and Limitations

This study had numerous strengths. As this study included all births in one state over a ten-year period, we were able to examine our outcomes of a large, diverse patient population and examine this over time. Given the large sample size, we could perform stratified analyses and examine outcomes by Medicaid type. It is important to note that given Emergency Medicaid recipients restricted access to care, they are likely systematically underdiagnosed for chronic medical conditions.

However, as this is a large dataset obtained from birth certificates and hospital discharge data, our ability to look at specific clinical circumstances was limited. For example, while we can speculate whether an ED visit was unnecessary and could have been managed on an outpatient basis, we are unable to determine this for sure as we could not assess individuals’ unique clinical circumstances. Future research should explore the reasons for the increase in use of the ED during pregnancy. Furthermore, we were unable to assess for differences in ED use by geographic location within the state and access, which could have been different between populations. Also, given missing data for race/ethnicity, we were unable to fully represent the impact on racial and ethnic groups.

## Conclusion

In this large study of pregnant patients with Medicaid insurance, we found there were significant differences by Medicaid type. We also found higher costs among those relying upon charity care, highlighting the potential economic impact of seeking emergency services during pregnancy.

## Figures and Tables

**Table 1:** Demographic and Clinical Characteristics of Medicaid Births with at least one ED Visit during Pregnancy in South Carolina by Medicaid Type 2010–2019 (n = 132,630)

	Traditional		Emergency		
	(N = 127,977)		(N = 4,653)		
Characteristics	N	%	N	%	*p-value*
**Maternal Age**					< 0.001
< 20	17,905	14.0	352	7.6	
20–24	51,784	40.5	1,106	23.8	
25–34	51,411	40.2	2,430	52.2	
≥ 35	6,877	5.4	765	16.4	
**Maternal Race/Ethnicity**					< 0.001
American Indian/Alaskan Native	577	0.5	19	0.4	
Asian	283	0.2	92	2.0	
Black	56,329	44.0	108	2.3	
Hawaiian/Pacific Islander	54	0.0	21	0.5	
Hispanic/Latina	1,706	1.3	2,653	57.0	
White	44,275	34.6	103	2.2	
Other/Unknown	24,753	19.3	1,657	35.6	
**Maternal Residential Location**					< 0.001
Urban	76,668	59.9	3,276	70.4	
Rural	35,062	27.4	851	18.3	
Missing	16,247	12.7	526	11.3	
**Multiparous**	77,885	60.9	3,491	75.0	< 0.001
**Body Mass Index**					
< 18.5	6,059	4.7	103	2.2	< 0.001
18.5–24.9	42,593	33.3	1,616	34.7	
25.0–29.9	29,918	23.4	1,547	33.2	
≥ 30.0	47,486	37.1	1,244	26.7	
Missing	1,921	1.5	143	3.1	
**No. of Prenatal Visits (Mean (SD))**	5.3	3.2	0.8	1.2	< 0.001
**Maternal Tobacco Use**	22,589	17.7	28	0.6	< 0.001
**Chronic Hypertension**	4,426	3.5	67	1.4	< 0.001
**Gestational Hypertension**	9,870	7.7	283	6.1	< 0.001
**Pre-pregnancy Diabetes**	1,614	1.3	86	1.8	< 0.001
**Gestational Diabetes**	6,917	5.4	425	9.1	< 0.001
**Gestational Age (weeks)**					< 0.001
< 28	1,011	0.8	23	0.5	
28–31	1,681	1.3	40	0.9	
32 – 26	12,838	10.0	379	8.1	
≥ 37	112,447	87.9	4,211	90.5	
**Cesarean Delivery**	44,060	34.4	1,381	29.7	< 0.001
**Intrapartum Severe Maternal Morbidity**	2,766	2.2	103	2.2	0.8096
**Obstetric Comorbidity Score (SMM) (Mean (SD))**	6.4	8.2	5.1	7.4	< 0.001
**Obstetric Comorbidity Score (non-Transfusion SMM) (Mean (SD))**	7.6	11.5	6.0	10.4	< 0.001

**Table 2:** Obstetric and Utilization Outcomes among Medicaid Births with at least One Emergency Department (ED) Visit during Pregnancy by Medicaid Type and Payor Category, South Carolina 2012–2019 (n = 132 630)

	Traditional (N = 127,977)		Emergency (N = 4,653)		
	Some or All Covered	All Self-pay/Charity	Some or All Covered	All Self-pay/Charity	p-value
	(n = 117,220; 91.7%)	(n = 10, 575; 8.3%)	(n = 1,936; 41.6%)	(n = 2,717; 58.4%)	
**Obstetric Outcomes**	**%**	**%**	**%**	**%**	
Cesarean Delivery	34.6	32.9	30.1	29.4	< 0.001
Pre-term Birth	12.1	12.6	10.4	8.9	< 0.001
NICU	8.5	8.8	5.9	6.4	< 0.001
Intrapartum SMM	2.1	2.3	2.4	2.1	0.5535
High Obstetric Comorbidity Index Score	22.3	22.7	19.4	16.9	< 0.001
**Utilization**					
Number of ED Visits during Pregnancy (Mean (SD))	2.5 (2.2)	1.4 (0.9)	1.8 (1.4)	1.4 (0.8)	< 0.001
Average Total Charges per ED Visit (Mean (SD))	$2,340 (1,930)	$2,270 (2,070)	$2,820 (2,550)	$2,800 (2,590)	< 0.001
Average Total Charges per ED Visit (Median (IQR))	$1,920 (1,920–2,930)	$1,780 (1,780–2,950)	$2,230 (2,230–3,450)	$2,330 (2,330–3,500)	< 0.001

Abbreviations: ED emergency department; NICU neonatal intensive care unit admission; SMM severe maternal morbidity; SD standard deviation; IQR interquartile range.

**Table 3:** Association between Medicaid Type and Emergency Department Visit during Pregnancy among South Carolina Medicaid Births, 2012–2019 (n = 132,630)

	At least 1 ED visit during pregnancy		At least 1 ED visit covered by Medicaid during Pregnancy	
	aOR	95% CI	aOR	95% CI
**Traditional**	*reference*		*reference*	
**Emergency**	0.26	(0.25–0.27)	0.14	(0.13–0.15)

Adjusted for maternal age and 5 prenatal visits flag

Abbreviations: ED *emergency department; aOR adjusted Odds Ratio*.

## Data Availability

The datasets used in the study are not publicly available.
